# Improving Human Activity Recognition Performance by Data Fusion and Feature Engineering

**DOI:** 10.3390/s21030692

**Published:** 2021-01-20

**Authors:** Jingcheng Chen, Yining Sun, Shaoming Sun

**Affiliations:** 1 Institute of Intelligent Machines, Hefei Institutes of Physical Science, Chinese Academy of Sciences, Hefei 230031, China; cjc324@mail.ustc.edu.cn (J.C.); ynsun@iim.cas.cn (Y.S.); 2 University of Science and Technology of China, Hefei 230026, China; 3 Chinese Academy of Sciences (Hefei) Institute of Technology Innovation, Hefei 230088, China

**Keywords:** feature selection, human activity recognition, activity of daily living, sensor fusion, wearable sensors, genetic algorithm, coordinate calibration

## Abstract

Human activity recognition (HAR) is essential in many health-related fields. A variety of technologies based on different sensors have been developed for HAR. Among them, fusion from heterogeneous wearable sensors has been developed as it is portable, non-interventional and accurate for HAR. To be applied in real-time use with limited resources, the activity recognition system must be compact and reliable. This requirement can be achieved by feature selection (FS). By eliminating irrelevant and redundant features, the system burden is reduced with good classification performance (CP). This manuscript proposes a two-stage genetic algorithm-based feature selection algorithm with a fixed activation number (GFSFAN), which is implemented on the datasets with a variety of time, frequency and time-frequency domain features extracted from the collected raw time series of nine activities of daily living (ADL). Six classifiers are used to evaluate the effects of selected feature subsets from different FS algorithms on HAR performance. The results indicate that GFSFAN can achieve good CP with a small size. A sensor-to-segment coordinate calibration algorithm and lower-limb joint angle estimation algorithm are introduced. Experiments on the effect of the calibration and the introduction of joint angle on HAR shows that both of them can improve the CP.

## 1. Introduction

Recently, with the rapid development of sensing, machine learning and micro-manufacturing technologies, the human activity recognition (HAR) and activity recognition system (ARS) have been greatly developed and applied in many fields, such as ergonomics, elder care, rehabilitation, internet of things, military, health monitoring, sports, etc. Basically, activity data from different dimensions are collected and analyzed to recognize and supervise different types of postures and motions. Therefore, recognition of activities of daily living (ADL), such as standing, sitting, squatting and walking, are particularly important for rehabilitations and health monitoring. For example, in fall detection, it is important to recognize and predict falls in real time from a large number of normal ADLs [1,2,3]; in artificial limbs and exoskeletons, it is necessary to identify human movement intentions and amplitudes to guide the movement of robots [4,5,6]; and in the disease monitoring and assessment, non-interventional identification of ADLs is essential because the extraction of motion characteristics of autonomous activities at home or in the community is beneficial for obtaining sufficient information on disease development [7,8,9].

At present, a variety of HAR technologies have been developed according to the different types of sensors and devices, for instance, wearable sensors, videos, and smartphones [10]. Compared with video, wearable devices are portable, low cost, have better privacy and little interference to users, which makes them applicable to non-laboratory environments and an appropriate solution for regular remote monitoring and guidance of rehabilitation. In addition, compared with smartphones, wearable sensors have better accuracy and robustness due to their more stable installation and positioning.

Two major processes are contained in HAR, which are, data processing and activity recognition. To be specific, data processing includes data acquisition, preprocessing, segmentation, feature calculation and selection. While activity recognition includes classification and evaluation. The HAR begins with collecting the motion information and bio-signals via variety of sensors, the most commonly used of which are accelerometer (ACC), gyroscope (GYR), magnetometer (MAG), pressure sensor, cardiotachometer and surface Electromyogram (sEMG). For example, quite a few studies use independent inertial measurement unit (IMU, which consists of ACCs and GYRs, sometimes MAGs), sEMG or IMU embedded in smart devices (e.g., smart bracelets) to identify multiple activities. According to the different sensor locations, these studies have achieved good recognition performances for different movements, such as gestures [11,12,13,14], upper limb movements [15], lower limb movements, comprehensive ADLs [16,17] and complex activities consisting of multiple movements.

In addition to using one kind of sensor or several homogeneous sensors (e.g., using ACCs and GYRs for rotation information), data fusion from heterogeneous sensors are continuously being developed to improve the accuracy of ARS. By collecting and filtering information from different physical dimensions, the impact of errors from homogeneous information is reduced [10]. For instance, Ai Qingsong et al. [18] fused the sEMG and ACC signals to identify the motion patterns of lower limbs and prove that the fused feature-based classification outperforms classification with only homogeneous sensors. Jia and Liu [19] achieved 99.57% accuracy in HAR by fusing ECG and ACC sensors, so that they could not only improve the recognition performance but also monitor more physiological signals of human activity. Meanwhile, Md. Al-Amin et al. [20] used a multimodal sensor system (which consists of 16 sEMG sensors, two 3-axis ACC, two 3-axis GYR, two 3-axis MAG and a Kinect sensor) to recognize worker actions in performing complex manufacturing tasks, and reached better performance than recognition using single type of sensor. For similar activities, walking and ascending/descending stairs, for instance, that are difficult to distinguish by using only one type of sensor, Lara et al. [21] noted that the fusion of vital signs and IMUs can greatly improve the performance of recognition of descending stairs. Furthermore, several studies have proposed technologies to fuse multimode sensors for both HAR and health-related applications, which include disease management, health status monitoring and disease assessment [7,22,23,24].

In terms of features, researchers prefer to extract features from the raw time-serial data rather than using the data itself for recognition. These features can be divided into two kinds [10]: one is the hand-extracted shallow features such as time-domain (TD), frequency domain (FD) and time-frequency domain (TFD) features; the other is the deep features automatically obtained by using artificial intelligence tools such as principal component analysis, independent component analysis and deep learning. Compared with the latter, although the former requires a large amount of labelled data to select features through a priori heuristic learning, it has smaller computation complexity for feature calculation and classification. To be specific, deep learning-based feature extraction can automatically learn feature representation from big data and achieve good accuracy, yet including millions of parameters or even more [25,26], which creates additional storage and computing requirements. Therefore, on the basis of ensuring the recognition performance, the shallow features may be more suitable for real-time applications with low power consumption, especially for real-time applications that need to extract corresponding features in the subsequent works, such as automatic disease assessment or health monitoring based on identification of specific activities. At present, most approaches extract the shallow features in IMU or sEMG for HAR based on the recommendation of the literature. Meanwhile, some studies discussed the influence of sensor and feature selection in activity recognition, and presented feature subset selection methods [27,28,29]. However, most of them focus on homogeneous sensors, and the selection results of feature subsets tend to end at a large scale. Besides, in addition to extracting features directly from the raw data, IMUs can also be used to calculate the rotation of the limbs [30,31]. Similar to the video-based method, this can be used to calculate the kinematic information, joint angles, for example, as a supplement for HAR without increasing the number of sensors and complexity of ARS. Particularly, it is worth mentioning that the method used to acquire the kinematic information of limbs by fusing the IMU data can also be used to correct the coordinate system deviation caused by the unrepeatable and inaccurate installation of sensors and devices, which are commonly exist. However, most HAR studies rarely describe the alignment approach of IMUs or do not align them.

The present study proposes a genetic algorithm-based feature selection algorithm with fixed activation number (GFSFAN) to select appropriate feature subsets for HAR with small scales and good classification performance. Besides, a sensor-to-body segment coordinate calibration algorithm for IMU is proposed to eliminate errors caused by the coordinate misalignment, and a lower limb joint angle estimation algorithm is introduced as well to expand the source of human motion information used in HAR. The experimental results indicate that (i) the proposed feature selection algorithm can achieve good classification performance with small feature subsets of manually set size, and (ii) the calibration algorithm and fusion of multiple heterogeneous sensors are both beneficial for improving HAR performance.

## 2. Algorithm Description

### 2.1. Calculation of the Joint Angle

Joint angles are calculated by fusing the acceleration and angular velocity data from the adjacent body segments, which are lower back (LB), left thigh (LT) and left shank (LS) in this work. Magnetometers are abandoned because of the uncertain magnetic field changes caused by concrete reinforced and metalware. In the proposed method, orientation of sensors are represented by quaternions, initialized by setting the 30 s standing phase at the beginning to be the initial state and updated through an attitude estimation algorithm presented by Mahony [32]. In addition, a sensor-to-segment coordinate calibration algorithm is required to eliminate errors caused by the unprecise and poorly repeatable alignment between the segment reference frame (SMRF) and the sensor reference frame (SSRF) [33,34].

**Figure 1:**
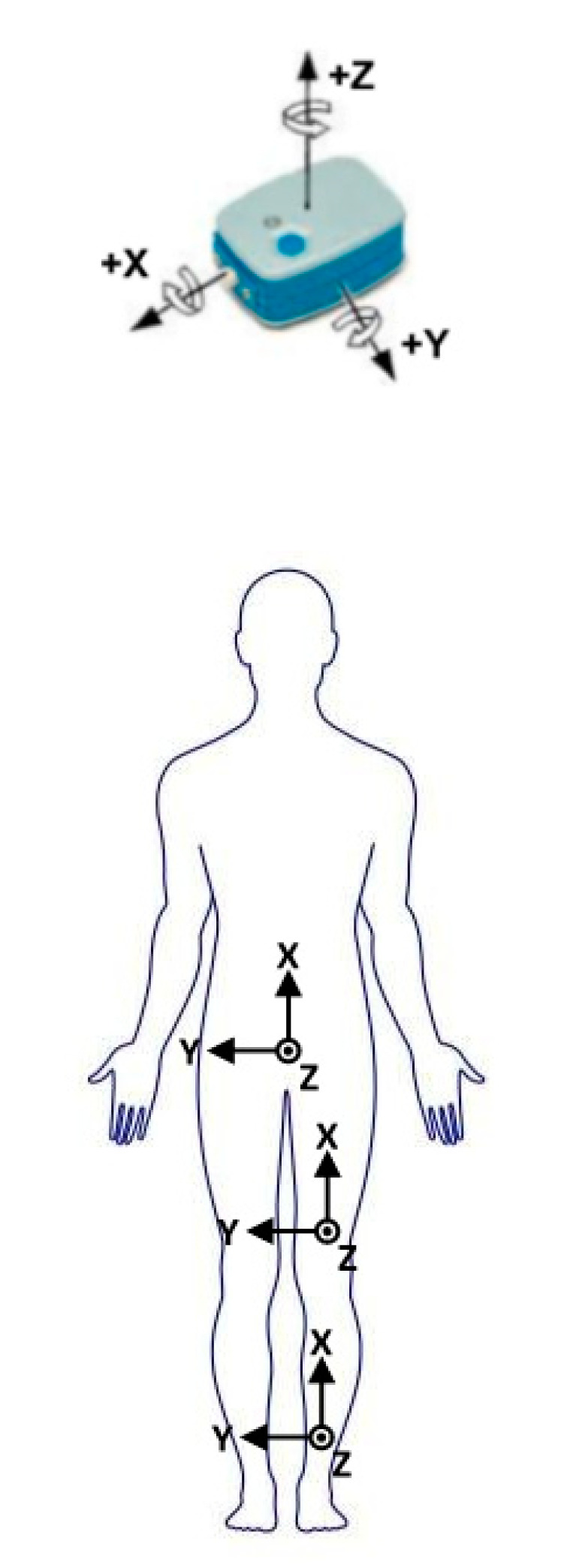
The segment reference frames and sensor reference frames.

The hip/knee joint can be modeled as a three-/one-dimensional joint connected two rigid segments; the knee joint is modeled as a one-dimensional joint because the range of motion in the flexion and extension direction is much larger than that in the other two directions. A set of SMRF and SSRF are defined as the right-handed Cartesian coordinate systems, as shown in Figure 1. The SMRF is defined as *X*-axis points up from feet to head in vertical axis, *Y*-axis points to medial in frontal axis and *Z*-axis points to forward in sagittal axis. Meanwhile, the SSRF is defined by the manufacturer.

**Figure 2:**
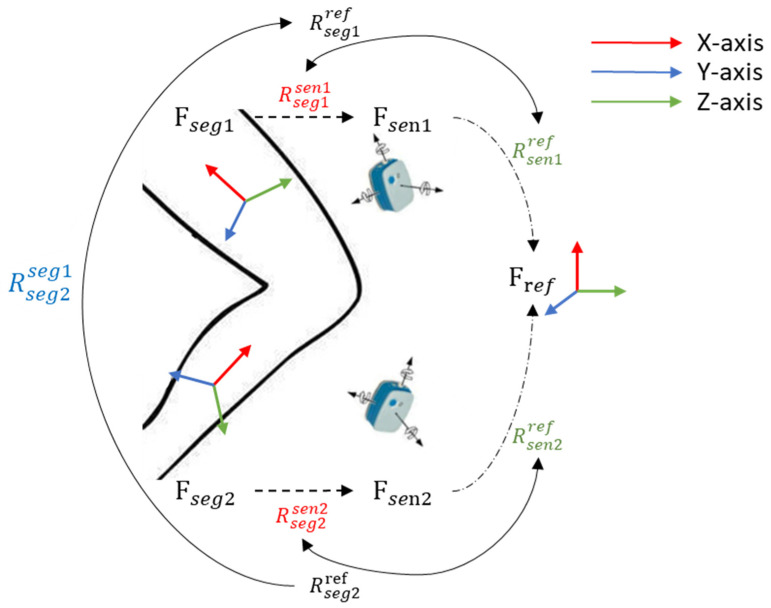
Schematic of joint angle calculation.

Figure 2 shows the schematic of joint angle calculation by taking the knee joint as an example. $F_{seg1}$, $F_{seg2}$, $F_{sen1}$ and $F_{sen2}$ represent the reference frames of different segments and sensors (seg1 and seg2 are left thigh and left shank, sen1 and sen2 are the Ultium sensors mounted on the corresponding segments). $F_{ref}$ represents the reference frame defined by the initial state, that is, all of the SMRF are set as unit matrix at that moment. Joint angle can be calculated as:

(1) $$A_{i}=\arccos\frac{{j_{1}}^{T}j_{2}}{\left\|j_{1}\|\|j_{2}\right\|}$$

where $j_{1}$ and $j_{2}$ denote the orientation of the two segments, with $j_{1}$ defined as the coordinate axis and $j_{2}$ calculated from the relative rotation between $F_{seg1}$ and $F_{seg2}$:

(2) $$R_{seg2}^{seg1}={\left(R_{seg1}^{ref}\right)}^{T}\times R_{seg2}^{ref}$$

where $R_{B}^{A}$ represents the orientation matrix of frame B with respect to frame A. $R_{segi}^{ref}$ (i = 1,2), the orientation matrix of segment frame with respect to the reference frame, is calculated by:

(3) $$R_{segi}^{ref}=R_{seni}^{ref}\times R_{segi}^{seni}$$

where $R_{seni}^{ref}$ can be transformed from the quaternion of the sensor, and $R_{segi}^{seni}$ is computed by figuring out the representation of the rotation axis of the segment (the coordinate axis of the SMRF) in the SSRF, as we assume that the relative orientation between the sensor and corresponding segment remains invariant while the sensor is fixed, though slight errors may be introduced by the displacement of soft tissue. Firstly, $x_{segi}^{seni}$ of all segments and $z_{segi}^{seni}$ of low back are computed by measuring the gravitational acceleration vector for corresponding segments in the 30 s standing phase and lying phase, respectively. Particularly, $y_{segi}^{seni}$ of thigh and shank, which are regarded as the representation of the knee flexion/extension axis in local reference frame, are computed by using an self-calibrating optimization algorithm proposed by Thomas Seel [35,36]. Subsequently, the third rotation axis of each segment is calculated by taking the cross product of the other two axes:

(4) $$z_{segi}^{seni}=x_{segi}^{seni}\times y_{segi}^{seni},\;\mathrm{or}\;y_{segi}^{seni}=z_{segi}^{seni}\times x_{segi}^{seni}$$

Besides, an orthogonalization process is introduced to correct the possible deviation of the second axis. Finally, the orientation matrix $R_{segi}^{seni}$ is obtained as:

(5) $$R_{segi}^{seni}=[x_{segi}^{seni},y_{segi}^{seni},z_{segi}^{seni}]$$

In addition to calculating the joint angle, $R_{segi}^{seni}$ is also used to correct all the IMU data as mentioned before. Two datasets were generated from the same original data: one with calibration and the other without calibration. The same subsequent processes were used for both datasets and the results of classification under different conditions (e.g., different ADLs, feature subsets, classification algorithms or sensor combinations) were obtained to verify the effect of calibration algorithm on the HAR performance.

### 2.2. Features

#### 2.2.1. Feature Calculation

The processed data are a set of labelled time series with fixed length, each series contains a large amount of sensor data. By extracting high-order features from each series, the data dimensions can be greatly reduced and the system robustness and classification accuracy can be improved. In this work, several widely used TD features, FD features and TFD features are selected to figure out the optimal or suboptimal feature subset for ADL classification.

- TD Features

The selected TD features are Mean Value (MV), Standard Deviation (SD), Variance (VAR), Root Mean Square (RMS), Skewness (SKE), Kurtosis (KUR), Interquartile Range (IQR), Peak to Peak(P2P), Mean Absolute Value (MAV), Zero Crossing (ZC), Waveform Length (WL), Slope Sign Change (SSC), Wilson Amplitude (WAMP), Log Detector (LD), 4-th order Auto Regressive Coefficient (ARC), Energy and Modified Mean Absolute Value (MMAV). In addition, Jerk, Correlation Coefficient (CC) and Signal Magnitude Area (SMA) are also calculated.

- FD Features

Fast Fourier Transform algorithm (FFT) is used to transform the raw time series data x(t) of each epoch into FD data p(f). Based on this, we extract several FD features, which are Mean Power Frequency (MPF), Median Frequency (MDF), One Quarter of Frequency (F25), Three Quarters of Frequency (F75), Top 3 Largest Value of DFT (3LVD) and Entropy.

- TFD Features

**Figure 3:**
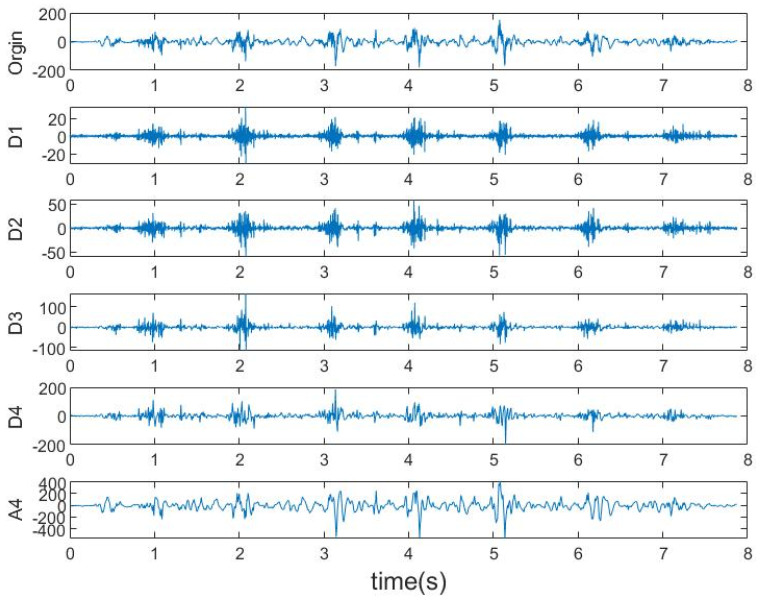
An example of wavelet decomposition of one sequence of LRF signal during walking.

Wavelet transformation (WT) is a commonly used TFD analysis tool in biomedical signal processing [37,38]. It decomposes signals into two sets of data, detail D[k] and approximation A[k]. To achieve optimal performance, a suitable wavelet function should be employed. According to the recommendations of previous reports [18,39,40,41], we adopted the sym4 mother wavelet as the wavelet basis at fourth decomposition level. An example of wavelet decomposition of WT is shown in Figure 3.

The selected TFD feature is Energy of Wavelet Coefficient (EWC), which is defined as the Energy of the final approximation and all four details.

The mathematical definitions of selected features are shown in Table 1. Where N and n denote the number of samples of raw data and number of sampling points of FFT, the $a_{i}$ and $e_{k}$ in ARC represent the AR parameters and white noise, while $Q_{1}$ and $Q_{3}$ in IQR are the first and third quartile of the raw signal. Besides, the threshold $\delta_{z}$, $\delta_{s}$ and $\delta_{w}$ are set to reduce the noise for computing ZC, SSC and WAMP [14,18,42,43,44].

All features are normalized with Min–Max Normalization. The initial feature sets of each sensor type are shown in the Table 2, 776 features are selected. To remove redundancy of the feature sets, we selected the suboptimal feature subset for ADL classification with method presented in Section 2.3.

#### 2.2.2. Resampling

Noticing that the mean value of ratio of the largest class to smallest class for data with different window parameters is larger than 5 (6 for the parameters we used), taking the imbalanced raw data as training set can easily lead to the classification bias towards the majority class [45]. Existing solutions include methods at data level and algorithm level; in this paper, we select the most commonly used data-level method: resampling. Specifically, the minority class is oversampled through the ADASYN algorithm proposed by Haibo He [46], while the majority class is undersampled by random sampling with replacement. The deviation caused by random algorithm is eliminated by an ensemble algorithm. Specifically, k times undersampling operations are performed firstly, then each set of the undersampling data is combined with the oversampling data calculated by ADASYN algorithm, respectively, to obtain k groups of resampled data. All subsequent algorithm operations run once for each group of resampled data, and the results are represented by the mean value of k results. Particularly, the resampling scale are set equal for each class and calculated as the geometric mean of the largest and smallest class. K is set to 10 in this study.

#### 2.2.3. Feature Evaluation

A class separability index is needed to preliminary evaluate the appropriateness of each feature. Several kinds of index were established in previous study, such as FI, Relief-F, Bhattacharyya Index, Chernoff Index, Divergence and Entropy. Among them, FI is used in this work due to the simplicity and stability. FI is computed by dividing the trace of the between-class scatter matrix by the trace of the within-class scatter matrix. The higher FI is, the better of the class separability of the feature.

The between-class scatter matrix $S_{b}$ and within-class scatter matrix $S_{w}$ are defined as:

(6) $$S_{b}=\overset{k}{\underset{i=1}{\sum }}p_{i}\left(m_{i}-m\right){\left(m_{i}-m\right)}^{T}$$

(7) $$S_{w}=\overset{k}{\underset{i=1}{\sum }}p_{i}E\left(\left(x-m\right){\left(x-m\right)}^{T}\right)$$

where $m_{i}$ is the mean of i-th class and m is the mean of all classes, while $p_{i}$ is the prior probability of i-th class. FI is defined as:

(8) $$FI=\frac{trace\left(S_{b}\right)}{trace\left(S_{w}\right)}$$

### 2.3. Feature Selection

Raw feature vector is multidimensional data with a lot of redundant and irrelevant information, methods that are commonly used for feature reduction include feature selection and feature extraction. Considering that feature extraction usually produces new features calculated by combining multiple raw features, which may increase the system burden in real-time use, feature selection is adopted.

**Figure 4:**
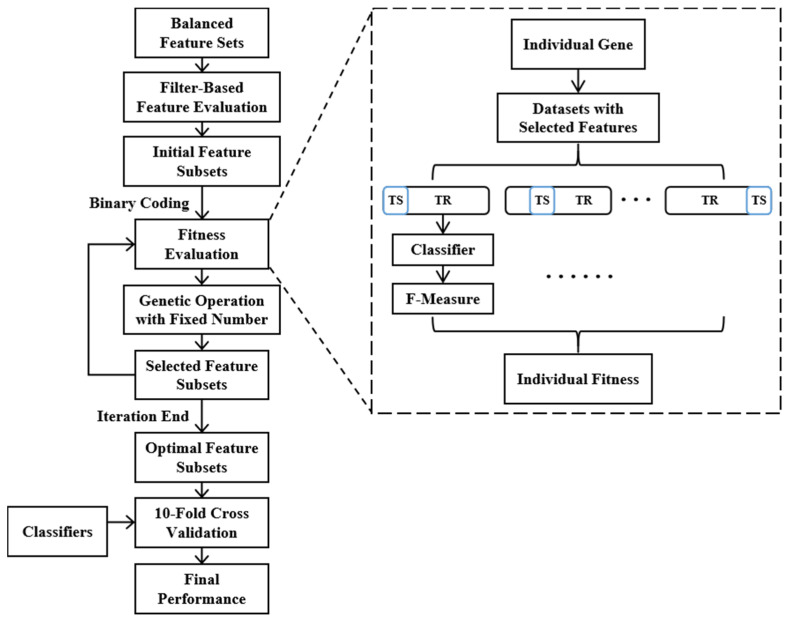
Block diagram of the two-stage genetic-based feature selection method.

Feature selection methods consist of filter-based, wrapper-based and embedded-based algorithm [47]. The evaluation criteria of filter-based feature selection are independent of the learning algorithm and obtained directly from the data set. Due to its high efficiency, the filter-based method is suitable for large-scale data set. However, searching for feature subsets related to classes and searching for feature subsets of optimal classification performance are two different tasks [48]. The wrapper-based method takes the performance of the learning algorithm as evaluation criterion and adopts a searching technology for feature selection. Compared to the filter-based method, the wrapper-based method is more accurate but less efficient. In the embedded-based method, feature selection algorithm is embedded into the learning process. Therefore, in order to meet the requirements of embedded algorithm, the learning algorithm suitable for embedded-based method is less than that for the wrapper-based method. To combine the advantages of filter-based and wrapper-based method, a two-stage feature selection algorithm is introduced in this work, as shown in Figure 4.

Firstly, a filter-based method is adopted to generate an initial feature subset rather than to eliminate some of the poorly performing features; therefore, it can not only expedite the wrapper-based search process, but also avoid the accidental deletion of important information. Specifically, all features are ranked by FI from most to least and given the probabilities of being selected into the initial feature subsets. The selection probability (SP) is set as: SPs of features with largest and smallest FI are P1 and P2, respectively, SPs for the remaining features in the rank are calculated according to the arithmetic sequence rule. P1 and P2 are set to 0.8 and 0.4, respectively. Subsequently, a wrapper-based method based on genetic algorithm (GA) is introduced. The reason for choosing GA algorithm among existing methods is because it is suitable for large-scale problems and has a better chance to find optimal solutions [49]. The full process of GA-based wrapper method is described as follows:

1. Initialization

Binary coding is used, and each bit of the individual represents one of the raw features, where “1” means selected and “0” means unselected. Each individual is initialized by weighting random sampling without replacement presented by Efraimidis and Spirakis [50], the $N_{init}$ features are selected with the largest $N_{init}$ weights, where the weight $w_{i}$ is calculated as:

(9) $$u_{i}=random\left(0,1\right),w_{i}=u_{i}^{1/SP_{i}}$$

Considering the system burden and performance requirements, the population size and the maximum number of iterations are set to 80 and 100, respectively.

2. Fitness evaluation function

The most important two factors that affect fitness of an individual are feature number and classification performance of the feature subset. Some studies integrated the two factors into one fitness evaluation function which combines a monotone decreasing function about feature number with a monotone increasing function about classification performance by a weight coefficient [49,51]. However, it is difficult to choose an appropriate weight coefficient in practical application and many of the results end up at large feature numbers. We assume that when the feature number of the optimal feature subset increases within a threshold (some point where redundancy is as important as new information), the classification effect and the system burden will both increase. Therefore, we propose the GFSFAN to meet the different requirements in practical applications. Where the “activation” denotes the feature is selected and its genetic locus in the individual is set to “1”. In this case, the fitness of an individual is calculated as follows:

Firstly, the selected data group is generated by combining the resampled data groups and the selected feature subsets defined by the individual. Then, Linear Discriminant Analysis (LDA) and Naive Bayes (NB) are applied to test the classification performance of the individual. In addition, the 10-fold cross validation is used to avoid overfitting. That is, each group of the selected data are randomly divided into ten folds, and each time, one of all folds is used as test set with the remaining nine folds used for training. The classification performance is then determined by averaging the F-measure (FM) for all folds of all resampled data groups. The FM is computed as the weighted harmonic average of Precision and Recall:

(10) $$P_{i}=\frac{n_{ii}}{\sum_{j=1}n_{ji}}$$

(11) $$R_{i}=\frac{n_{ii}}{\sum_{j=1}n_{ij}}$$

(12) $$FM_{i}=\frac{2P_{i}R_{i}}{P_{i}+R_{i}}$$

where $n_{ij}$ represents the number of samples identified as class j and actually class i. Finally, the fitness of an individual is computed by:

(13) $$f=\exp\left(FM_{all}\right)+\alpha \;\exp\left(FM_{min}\right)$$

where $FM_{all}$ and $FM_{min}$ are the mean and minimum FM of all classes, respectively. The weight coefficient α is set to 0.1 to make the overall classification performance as the main part of evaluation, and the worst classification performance in all classes as a supplement when the overall classification effect is similar.

3. Genetic operators

Tournament selection operator, crowding method and adaptive probability based approach are used to prevent the premature convergence [52]. Where the adapting probabilities are determined by the fitness of the individual, as well as the upper limits and lower limits of the probability:

(14) $$p=\{\begin{array}{r} p_{1}+\left(p_{2}-p_{1}\right)\frac{f_{max}-f}{f_{max}-\bar{f}},\;\;f\geq \bar{f}\\ p_{2},\;\;f<\bar{f} \end{array}$$

where the probability range [$p_{1}$,$p_{2}$] is set as [0.4,0.8] for crossover operation and [0.1,0.4] for mutation operation, $f_{max}$ and $\bar{f}$ are the maximum and mean fitness in the population, respectively. In particular, f is the larger fitness of the two parents in crossover operation.

A crossover operator with fixed activation number (COFAN) and a mutation operator with fixed activation number (MOFAN) are proposed to ensure that the feature activation number of each individual in the genetic operation is equal to the initial activation number $N_{init}$.

- COFAN: Firstly, the gene bits activated in each parent individual are extracted to form an intermediate individual of length $N_{init}$, each of whose gene bits represents the sequence number of an activated gene bits in the parent. Then, the same genes were selected from the intermediate individuals of the two parents to form the homogeneous gene pair, and remaining gens of each intermediate individuals are made into the heterogeneous gene pairs. The two-point crossover operation is performed on the two heterogeneous gene pairs to produce the progeny heterogeneous gene pairs, which are then combined with the homogeneous gene pair to form progeny intermedia individuals. Finally, each of the children is produced by setting the corresponding gene bits of an unactivated individual to “1” according to the progeny intermedia individual.
- MOFAN: Mutation operation is performed on each activated gene bits of the parent individual according to the mutation probability and the actual number of mutations is recorded firstly. Subsequently, the same number of unactivated bits are randomly selected to perform the mutation operation and the child individual is finally generated.

Six widely used classifiers are applied to test the classification performance of the selected feature subsets: Center-Nearest Neighbors, K-Nearest Neighbors (KNN), LDA, NB, Random Forests (RF) and Linear Support Vector Machine (SVM). Specifically, K is set to 1 in KNN, the tree number is set to 8 in RFs and the one-against-the rest strategy is used in SVM in the present study. Five groups of feature subsets with different fixed numbers (five features, eight features, 10 features, 15 features and 20 features) are generated with the proposed GFSFAN. The aim of this process is to verify the performance of the proposed approach in case of different system burden requirements. Besides, filter-based and wrapper-based selection processes are implemented as comparisons. The filter-based process is a two-stage algorithm: features not relevant to the classification are removed by a Relief-F algorithm, firstly, and then the correlations between remaining features are calculated to remove the redundancy. The criterion for removing redundant features is that feature with highest Relief-F value is retained among several strongly related features. The wrapper-based process is a Sequential Forward Selection (SFS) algorithm [53,54], the evaluation criterion of the wrapper process is the FM of selected feature subsets with LDA and NB.

## 3. Experiments Description

### 3.1. Participants

Thirteen healthy male participants and four female participants were recruited in this study. The participant characteristics are presented in Table 3. All participants were screened according to the following criteria: (1) healthy adult with no myopathies, orthopedic or metabolic disease or injury within six months before the tests start; (2) has no or litter previous experiences in relevant experiments. Participants were informed of the benefits and risks of the study prior to signing the informed consent. All experiment procedures covered in this study were evaluated and approved by the Ethic Committee of the Affiliated Hospital of Institute of Neurology, Anhui University of Chinese Medicine, which is the project partner of the fund supporting this research. The approval of the ethics committee is in accordance with the guidelines of Declaration of Helsinki and the National law of China.

### 3.2. Data Collection

#### 3.2.1. Activities and Procedure

**Figure 5:**
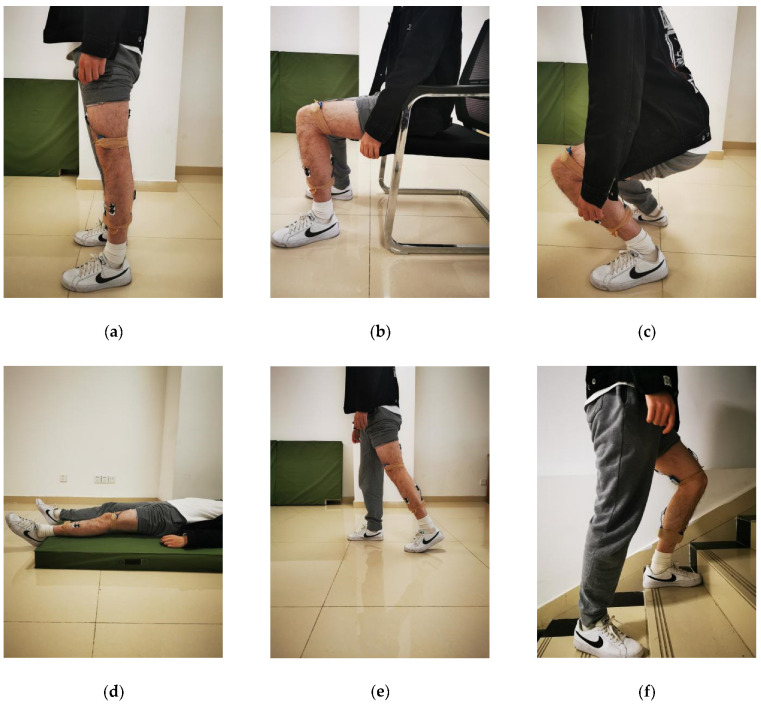

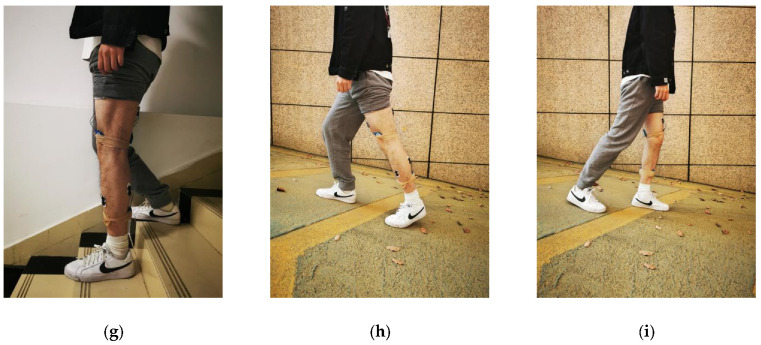
The selected ADLs: (**a**) standing, (**b**) sitting, (**c**) squatting, (**d**) lying, (**e**) walking, (**f**) ascending stairs, (**g**) descending stairs, (**h**) ascending ramps, and (**i**) descending ramps.

Walking (WLK), ascending stairs (AS), descending stairs (DS), ascending ramps (AR), descending ramps (DR), standing (STD), sitting (SIT), squatting (SQT) and lying (LY) were examined in this work. The selected ADLs are shown in Figure 5.

Participants performed each activity according to the following standard: (1) WLK (10 m straight walking), AS/DS (10 conventional building stairs with 16cm height of each) and AR/DR (15 m slope of about 8 degrees) were performed at preferred speed. (2) During STD, participants were required to stand straightly with feet shoulder-width apart and look ahead. (3) Upper limbs were not allowed to exert force while SIT or SQT. (4) Hold all four postures (STD, SIT, SQT and LY) at least 3 s each time.

The tasks were divided into three parts and carried out in three adjacent places at the same laboratory building. WLK and all of the four postures were carried out in the activity lab provided by the Research Center for Information Technology of Sports and Health. During the experiments, participants randomly performed activities and completed each activity at least 10 times. AS/DS and AR/DR were carried out at least 15 min each time on the staircase and garage ramp, respectively. Before starting each part, participants were requested to stand still for 30 s. Specially, a 3 s standing period between each activity was required to allow video tagging. All wearable sensors could not be disassembled or moved after installation until all activities are completed.

Participants were informed to report feeling tired or unwell at any time during the experiments, the tests would then be suspended until they recover or postponed. Before the formal experiments began, participants were required to be familiar with the criteria and procedures under the guidance of the experimenter. Eight participants completed a total of 10 experiments within one week.

#### 3.2.2. Sensors Configuration and Data Acquisition

Muscle activities were recorded by NORAXON Ultium wireless sensors (Noraxon, Scottsdale, AZ, USA) at 2000Hz. The Ultium sensors, which were placed on the skin over osseous structures or tendons, are reference electrodes as well as EMG signal processing and wireless transmission devices. Disposable, self-adhesive Ag/AgCL dual electrodes were placed on the skin over the belly of the target muscles along the major orientation of muscle fibers. The dual electrode is a pair of electrodes with 2 cm apart, center to center. Before positioned the electrodes or Ultium sensors, participants’ skin over the target muscles was cleaning with an alcohol-soaked pad to keep the impedance of the skin low and stable. The target muscles are left rectus femoris (LRF, near the midline of the left thigh, approximately halfway between the ASIS and the proximal patella), left semitendinosus (LSEM, on the medial aspect of the left thigh, located approximately 3 cm from the lateral border of the thigh and approximately half the distance from hip to the back of the knee.), left tibialis anterior (LTA, lateral to the medial shaft of the left tibia, at approximately one-third the distance between the knee and the ankle), and left lateral gastrocnemius (LLGA, lateral to the back of left shank, at approximately half the distance between the back of knee and heel).

Motion information of trunk and lower limbs was collected by MPU9250 (Invensense, San Jose, CA, USA) embedded in Ultium sensors at 200 Hz. The Ultium sensors which acted as IMUs were installed at L5/S1 of the spine at LB, at the distal end of the front of LT (2cm from the LRF electrodes) and at the distal end of the left tibia in the crus inside, which shared the same Ultium sensor as the reference electrode at LTA (LS). The *X*-axis of IMUs aligned the vertical axis pointing to the head direction and *Z*-axis aligned the sagittal axis pointing forward direction.

**Figure 6:**
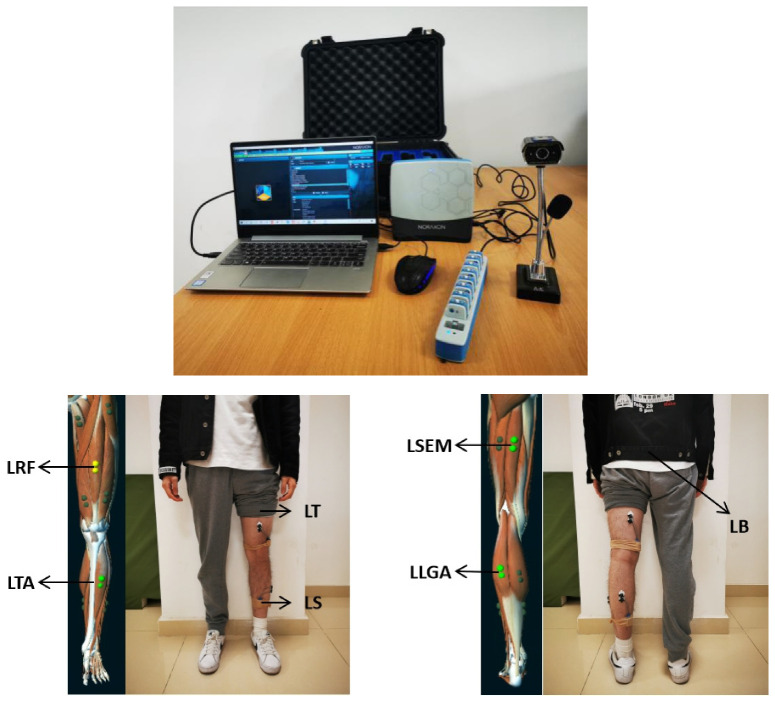
Experiment system and the arrangement of sensors.

All sensors were attached to the participant’s skin by double-sided adhesive tape and secured with a self-viscoelastic sports bandage. The experiment system and arrangement of sensors is shown in Figure 6.

Sensor data was sent via Bluetooth to the NORAXON Ultium Receiver, which was connected to a laptop via USB3.0 and synchronized with a 720P webcam for recording video. Raw data was collected and marked using the myoRESEARCH 3.12 (Noraxon USA). Two assistants who were not involved in the experiment were recruited, one marked the participants’ activities according to the synchronized video recording, the other checked the marked video and discussed with the first in case of disagreement.

### 3.3. Preprocessing and Segmentation

All signal processing were performed on the Matlab software (MATLAB R2018a, The MathWorks, Inc., Novi, MI, USA). Raw sEMG signals were filtered through a 4th order Butterworth 10–500 Hz bandpass filter and smoothed with a 50 ms moving average window. Raw IMU signals were elliptically corrected to remove the static error before using. Acceleration and angular velocity signals were filtered through an 8-order Butterworth 10 Hz lower filter and an 8-order Butterworth 30 Hz lower filter, respectively. The 30 s standing phase was used for normalization, which was dividing all acceleration data by the average resultant acceleration and subtracting all angular velocity data from the average angular velocity over the 30 s standing phase. Posture information was calculated by fusing the acceleration and angular velocity data from both sides of the target joints, which is described in Section 2.1.

The processed data were then segmented into epochs by a moving window to extract features. In this case, the window width and overlap rate play great roles in classification. For instance, a window with larger width may contains more than one activity and reduces the accuracy of classification, while small window width may result in insignificant features and overburdened systems.

Different window parameters were set to figure out the appropriate ones, the criteria for the window parameters to be selected are: (1) the increment of moving window should be lower than 300ms to avoid introducing perceivable delay [55]; (2) the overlap rates were 50%, 60%, 70%, 75% and 80%; (3) window widths were set as integer multiples of 80ms (160 sample points for EMG and 16 sample points for IMU). Fisher Index (FI), which is described in detail in Section 2.2.3, were used to evaluate the class separability of features. Specifically, summations of Fisher Index (SFI) of different sets of features distinguished by different window parameter pairs were calculated to evaluate the effects of these window parameters. Meanwhile, the Friedman test and Wilcoxon signed-rank test were used to check the influence of window parameters on the SFIs of all features. Finally, the most appropriate window parameter pairs were determined by analyzing these results and the rest of this work was based on these parameters.

## 4. Results

**Figure 7:**
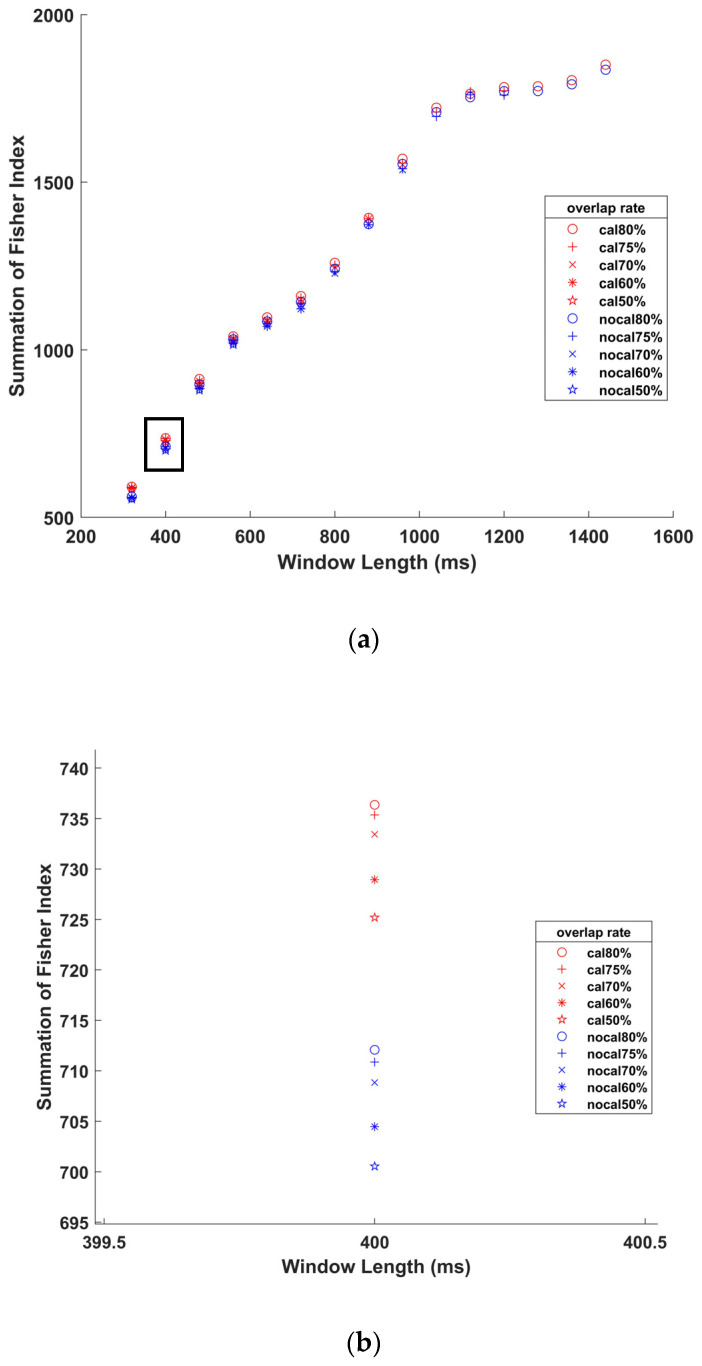
(**a**) SFI of all features with different window parameter pairs with and without calibration of IMUs. (**b**) Enlarge details of the black box in (**a**) at the window length of 400 ms. Where red means calibrated and blue means uncalibrated.

Figure 7 shows the SFIs of all features with different window parameter pairs. The purpose of this diagram is to figure out the most appropriate window parameters. The significance probability of Friedman test on all the five overlap rates and significance probabilities of Wilcoxon signed-rank tests on each pair of overlap rates are below 0.05. In Friedman test, the average ranks of SFIs distinguished by the five overlap rates are sorted from largest to smallest as: 80%, 75%, 70%, 60% and 50%, which is the same as the order of average SFIs of the five groups. The most appropriate window parameters were selected and all the following results were calculated with the datasets generated with the selected window parameters.

**Figure 8:**
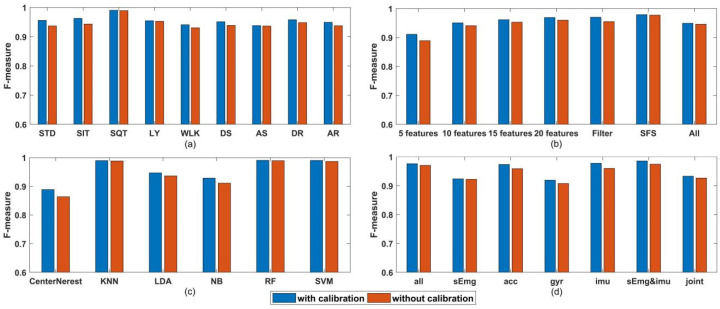
(**a**) F-measure per activity for dataset with calibration and without calibration. (**b**) F-measure per feature subset for dataset with calibration and without calibration. (**c**) F-measure per classification algorithm for dataset with calibration and without calibration. (**d**) F-measure per sensor combination for dataset with calibration and without calibration.

Effect of calibration on classification are shown in Figure 8. Mean FM derived from the classification of the two datasets described in Section 2.1 with different conditions are shown in the following order: Figure 8a shows mean FM of different activities, Figure 8b shows mean FM of different feature subsets, Figure 8c shows mean FM of different classification algorithms and Figure 8d shows mean FM of different sensor combinations. Significance probability of Wilcoxon signed-rank tests on FMs of classification on datasets with or without calibration at each condition is below 0.05. Additionally, in almost all conditions, FM of classification on datasets with calibration is higher than that without calibration.

**Figure 9:**
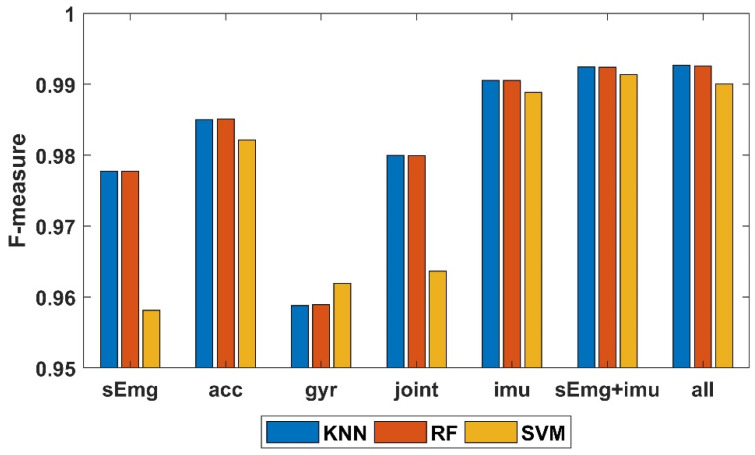
Mean F-Measure of all feature subsets per sensor combination and classification algorithms (KNN, RF and SVM).

**Figure 10:**
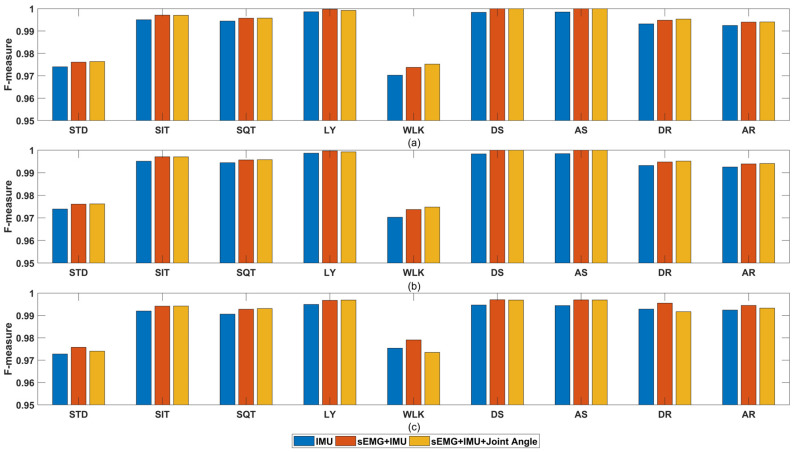
Mean F-Measure per activity and multiple sensors combination with different classification algorithms: (**a**) KNN; (**b**) RF; (**c**) SVM.

Figure 8c revealed that among all the classification algorithm used in this work, KNN, Random Forest and SVM end up with the best performance (their FM are 0.982, 0.982 and 0.976, respectively), which is similar with the result in [17]. Therefore, to simplify the diagram, only the three best classification algorithms are considered. The classification results of different sensor combinations and different feature subsets with KNN, RF and SVM are listed in Table 4. For GFSFAN only 10 features are listed as an example in the table. Next, mean FM of all feature subsets per sensor combinations and classification algorithms are shown in Figure 9. From this figure, the effect of different sensor combinations on the classification performance can be revealed. Particularly, mean FM of multiple sensors combinations per activity are shown in Figure 10.

All the feature selection and classification algorithms were implemented in Matlab R2018a, and the statistical processes were performed in SPSS 22.0 on a 3.0 GHz Inter(R) Core (TM) i5 processor with 8GB RAM. The segmentation and feature calculation steps required 50 min for the datasets with the selected window parameters. The resampling step required 37 s and the GFSFAN algorithm required at most 226 s (20 features for the combination of all sensors).

## 5. Discussion

The present study proposes a feature selection process from multiple types of wearable sensors to recognize nine activities of daily living. The purpose of this process is to reduce the system burden of ARS by significantly discarding the number of features, while keeping appropriate classification performance at the same time. Different classification algorithms with different sensor combinations were applied on the datasets generated from the experiment described above to analyze the influence of classifiers and sensor combinations on the classification performance. Besides, a sensor-to-body segment coordinate calibration algorithm for IMUs is proposed and the effect of this calibration process on the classification performance of HAR was also analyzed.

In Figure 7, the order of SFIs with different overlap rates from largest to smallest is: 80%, 75%, 70%, 60% and 50%, which can be seen in the enlarged detail in Figure 7b. Besides, the results of significance difference analysis on overlap rates show that the differences between each overlap rate are significance. Thus, SFI with 80% overlap rate is significantly higher than that with other overlap rates, although the differences are small. In addition, the highest overlap rate means the largest number of sliding windows, which is conducive to the classification. In Figure 7a, the SFI increases with the increase of window length, and this growth trend slows down greatly at 1040 ms. In sum, considering the separability of features and the number of windows, the window parameters selected in this work are 1040 ms of window length with an 80% overlap rate.

For the calibration process, the significance tests for classification results with or without calibration at each condition revealed that the former was significantly superior to the latter, which can also be seen in Figure 8, except for the sEmg features as the calibration algorithm only corrects the coordinate deviation of the inertial sensors.

In Figure 8b, the classification of ADLs can achieve acceptable results with the feature subsets obtained through the GFSFAN algorithm (the FM are 0.901 for 5 features, 0.941 for 10 features, 0.952 for 15 features and 0.955 for 20 features) compare to that through the Filter based feature selection (0.959) and SFS (0.969). Among them, the result is worst for 5 features, which may be due to the prematurity of GA process and information loss caused by the extremely small number of features. From Table 4, all three feature selection methods reduced the number of features, while also improve the performance of classification. Besides, the GFSFAN significantly discarded most of the features and generated small feature subsets with manually set size. With these features, the classification models building time and system burden for real-time HAR can be greatly lowered while the classification performance will not be adversely affected greatly.

In Figure 9 and Figure 10, among all the four single types of sensors, ACC achieved the best classification performance while GYR achieved the worst. Joint angle had the second highest FM as data fusion of ACC and GYR. As expected earlier, HAR with the combination of ACC and GYR (IMU) had significantly higher FM compared to that with only ACC or GYR. Furthermore, HAR with combination of sEMG and IMU showed slight improvement over HAR with the combination of IMU, and the improvement was also evident when comparing HAR with the combination of all sensors and HAR with combination of sEMG and IMU. However, this improvement was non-significant when joint angles are added. This may be due to the fact that joint angles are generated through IMU data fusion. Additionally, the positive impact of newly useful information provided by these new features may be almost equal to the negative impact of the increase in the number of features on the estimation of the optimal subset of features.

For classifiers, KNN, RF and SVM provide significantly higher performance, as well as longer training or predicting time than the CenterNearest, LDA and NB. In terms of classification performance, KNN and RF are the best of all, however KNN requires a large amount of memory to store the training sets and may be the slowest when dealing with large data. LDA and NB provide lower performance, yet not as low as CenterNearest, but are very fast. Thus, LDA and NB are appropriate as wrapper algorithms in the feature selection process and as the classifiers in case of limited computing resources.

The proposed feature selection algorithm for HAR can be also used in human health-related applications which may require compact ARS. For example, extracting features of users performing specific tasks on the basis of identifying and segmenting related activities to assess the disease progression is an important process towards automated disease monitoring. In this case, establishing ARS by using features that are also useful for subsequent assessing process with a few additional features can significantly reduce the system burden. Thus, according to the requirements of classification performance and system burden, it can be a good method to use the proposed algorithm to select the optimal feature subsets from various features for building ARS.

## 6. Conclusions

This study proposes a feature selection method that can obtain feature subsets for HAR as well as a sensor-to-segment coordinate alignment algorithm and a joint angle estimation algorithm. Comparing with other feature selection algorithms, the proposed algorithm can greatly discard features and maintain comparable classification performance. The sensor coordinate calibration algorithm is proved to be beneficial for HAR. Although some cases do not show significant improvement when sensor types are adding, comprehensive results indicate that the fusion of heterogeneous sensors (sEMG and Joint angel in this case) exhibits satisfactory performance for HAR. Six different classifiers are introduced to recognize human activities of daily living, and the results suggest RF and SVM appropriate classifiers for HAR, while using LDA and NB when memory and computing resources are limited. Adding more types of vital sign sensors and studying relevant open databases will be further explored in future research.

## Figures and Tables

**Table 1:** Mathematical definitions of features.

Feature	Mathematical Definition	Feature	Mathematical Definition
Mean Value (MV)	$\frac{1}{N}\sum_{i=1}^{N}x_{i}$	Standard Deviation (SD)	$\sqrt{\frac{1}{N-1}\sum_{i=1}^{N}{\left(x_{i}-\bar{x}\right)}^{2}}$
Variance (VAR)	$\frac{1}{N-1}\sum_{i=1}^{N}x_{i}{}^{2}$	Root Mean Square (RMS)	$\sqrt{\frac{1}{N}\sum_{i=1}^{N}x_{i}{}^{2}}$
Skewness (SKE)	$\frac{1}{N\sigma^{3}}\sum_{i=1}^{N}{\left(x_{i}-\bar{x}\right)}^{3}$	Kurtosis (KUR)	$\frac{1}{N\sigma^{4}}\sum_{i=1}^{N}{\left(x_{i}-\bar{x}\right)}^{4}$
Interquartile Range (IQR)	$Q_{3}-Q_{1}$	Peak to Peak(P2P)	$x_{max}-x_{min}$
Mean Absolute Value (MAV)	$\frac{1}{N}\sum_{i=1}^{N}\left|x_{i}\right|$	Waveform Length (WL)	$\sum_{i=1}^{N-1}\left|x_{i+1}-x_{i}\right|$
Zero Crossing (ZC)	$\{\begin{array}{c} ZC=\sum_{i=1}^{N}z_{i}\times zc_{i}\\ zc_{i}=sgn\left(-x_{i}x_{i+1}\right)\\ z_{i}=\{\begin{array}{l} 1,\;\left|x_{i}-x_{i+1}\right|>\delta_{z}\\ 0,\;else \end{array} \end{array}$	Slope Sign Change (SSC)	$\{\begin{array}{c} SSC=\sum_{i=2}^{N-1}s_{i}\times ssc_{i}\\ ssc_{i}=sgn\left[\left(x_{i}-x_{i-1}\right)\left(x_{i}-x_{i+1}\right)\right]\\ s_{i}=\{\begin{array}{l} 1,\;\left|x_{i}-x_{i+1}\right|>\delta_{s}\;\cap \;\left|x_{i}-x_{i-1}\right|>\delta_{s}\\ 0,\;else \end{array} \end{array}$
Wilson Amplitude (WAMP)	$\sum_{i=1}^{N-1}sgn\left(\left|x_{i+1}-x_{i}\right|-\delta_{w}\right)$	Log Detector (LD)	$\exp\left(\frac{1}{N}\sum_{i=1}^{N}log\left(\left|x_{i}\right|\right)\right)$
Auto Regressive Coefficient (ARC)	$x_{k}=\sum_{i=1}^{4}a_{i}x_{k-i}+e_{k}$	Energy	$\frac{1}{N}\sum_{i=1}^{N}{\left|x_{i}\right|}^{2}$
Modified Mean Absolute Value (MMAV)	$\{\begin{array}{c} \frac{1}{N}\sum_{i=1}^{N}\omega_{i}\left|x_{i}\right|\\ \omega_{i}=\{\begin{array}{l} 1,\;\;0.25N\leq i\leq 0.75N\\ 0,\;\;else \end{array} \end{array}$	Correlation Coefficient (CC)	$\frac{\sum_{i=1}^{N}\left(cca_{i}-\bar{cca}\right)\left(ccb_{i}-\bar{ccb}\right)}{\sqrt{\sum_{i=1}^{N}{\left(cca_{i}-\bar{cca}\right)}^{2}\sum_{i=1}^{N}{\left(ccb_{i}-\bar{ccb}\right)}^{2}}}$
Jerk	$\frac{1}{2}\sum_{i=2}^{N}\sum_{j=1}^{3}{\left(ACC_{j}\left(i\right)-ACC_{j}\left(i-1\right)\right)}^{2}$	Signal Magnitude Area (SMA)	$\frac{1}{N}\sum_{i=1}^{N}\left(\left|smax_{i}\right|+\left|smay_{i}\right|+\left|smaz_{i}\right|\right)$
Mean Power Frequency (MPF)	$\frac{\sum_{i=1}^{n}p_{i}f_{i}}{\sum_{i=1}^{n}p_{i}}$	Entropy	$-\sum_{i=1}^{n}\left(\frac{p_{i}}{\sum_{i=1}^{n}p_{i}}ln\left(\frac{p_{i}}{\sum_{i=1}^{n}p_{i}}\right)\right)$
Median Frequency (MDF)	$\sum_{i=f_{min}}^{MDF}p_{i}=\sum_{i=MDF}^{f_{max}}p_{i}$	One Quarter of Frequency (F25)	$3\sum_{i=f_{min}}^{F25}p_{i}=\sum_{i=F25}^{f_{max}}p_{i}$
Three Quarters of Frequency (F75)	$\sum_{i=f_{min}}^{F75}p_{i}=3\sum_{i=F75}^{f_{max}}p_{i}$		

**Table 2:** The initial select feature sets of each sensor data category.

Feature	sEMG	ACC	GYR	Joint
MV	●	●	●	●
SD	●	●	●	●
VAR	●	●	●	●
RMS	●	●	●	●
SKE	●	●	●	●
KUR	●	●	●	●
IQR	●	●	●	●
P2P	●	●	●	●
MAV	●	●	●	●
WL	●	●	●	●
ZC	●		●	
SSC	●		●	
WAMP	●		●	
LD	●	●	●	●
ARC	●	●	●	●
Energy	●	●	●	●
MMAV	●	●	●	●
CC		●	●	
Jerk		●		
SMA		●	●	
MPF	●	●	●	●
MDF	●	●	●	●
Entropy	●	●	●	●
F25	●	●	●	●
F75	●	●	●	●
Top 3 Largest Value of DFT (3LVD)	●	●	●	●
Energy of Wavelet Coefficient (EWC)	●	●	●	

CC of ACC and GYR denote the correlation coefficient of different axes in each sensor, such as CCxy, CCxz, CCyz.

**Table 3:** The participant characteristics (Mean ± Standard-Deviation).

Gender	Number	Age (Year)	Weight (kg)	Height (cm)
Male	13	27.5 ± 2.53	69.8 ± 7.65	176.5 ± 6.23
Female	4	29.3 ± 4.79	59.6 ± 4.03	159.8 ± 2.75
All	17	27.6 ± 3.10	66.8 ± 7.95	172.5 ± 9.17

**Table 4:** The classification performance of different sensor combinations and different feature subsets with KNN, RF and SVM.

		GFSFAN 10 Features		Filter		SFS		All Features	
Sensor Comb.	Algo.	FN	FM	FN	FM	FN	FM	FN	FM
**sEMG**	KNN	10	0.977	59	0.98	19	0.981	132	0.973
	RF		0.977		0.98		0.981		0.973
	SVM		0.933		0.957		0.984		0.960
**ACC**	KNN	10	0.985	114	0.984	36	0.986	285	0.985
	RF		0.985		0.984		0.986		0.985
	SVM		0.980		0.981		0.985		0.984
**GYR**	KNN	10	0.945	121	0.948	55	0.965	309	0.977
	RF		0.946		0.948		0.965		0.977
	SVM		0.943		0.949		0.978		0.977
**Joint Ang.**	KNN	10	0.993	20	0.993	34	0.994	50	0.934
	RF		0.993		0.993		0.994		0.934
	SVM		0.983		0.988		0.986		0.881
**IMU (ACC+GYR)**	KNN	10	0.987	231	0.991	31	0.992	594	0.991
	RF		0.989		0.991		0.992		0.991
	SVM		0.987		0.988		0.991		0.989
**sEMG+IMU**	KNN	10	0.993	289	0.994	37	0.994	726	0.988
	RF		0.993		0.994		0.994		0.988
	SVM		0.989		0.991		0.993		0.992
**sEMG+IMU+Joint Ang.**	KNN	10	0.994	307	0.994	36	0.995	776	0.988
	RF		0.994		0.994		0.995		0.988
	SVM		0.983		0.991		0.994		0.983
**Mean Value**	KNN	10	0.982	163	0.986	35.4	0.987	410	0.977
	RF		0.982		0.986		0.987		0.977
	SVM		0.971		0.977		0.987		0.966
